# Epigenomic Echoes—Decoding Genomic and Epigenetic Instability to Distinguish Lung Cancer Types and Predict Relapse

**DOI:** 10.3390/epigenomes9010005

**Published:** 2025-02-05

**Authors:** Alexandra A. Baumann, Zholdas Buribayev, Olaf Wolkenhauer, Amankeldi A. Salybekov, Markus Wolfien

**Affiliations:** 1 Department of Systems Biology and Bioinformatics, Institute of Computer Science, University of Rostock, 18051 Rostock, Germany; alexandra.baumann@uni-rostock.de (A.A.B.);; 2 Faculty of Medicine Carl Gustav Carus, Institute for Medical Informatics and Biometry, TUD Dresden University of Technology, 01069 Dresden, Germany; 3 Department of Computer Science, Faculty of Information Technologies, Al-Farabi Kazakh National University, 050040 Almaty, Kazakhstan; 4 Leibniz-Institute for Food Systems Biology, Technical University of Munich, 80333 Freising, Germany; 5 Stellenbosch Institute of Advanced Study, Wallenberg Research Centre, Stellenbosch University, Stellenbosch 7535, South Africa; 6 Regenerative Medicine Division, Cell and Gene Therapy Department, Qazaq Institute of Innovative Medicine, 010000 Astana, Kazakhstan; 7 Kidney Disease and Transplant Center, Shonan Kamakura General Hospital, Kamakura 247-8533, Japan; 8 Center for Scalable Data Analytics and Artificial Intelligence (ScaDS.AI), 01069 Dresden, Germany

**Keywords:** genomic instability, epigenetics, biomarkers, lung cancer, disease progression

## Abstract

Genomic and epigenomic instability are defining features of cancer, driving tumor progression, heterogeneity, and therapeutic resistance. Central to this process are epigenetic echoes, persistent and dynamic modifications in DNA methylation, histone modifications, non-coding RNA regulation, and chromatin remodeling that mirror underlying genomic chaos and actively influence cancer cell behavior. This review delves into the complex relationship between genomic instability and these epigenetic echoes, illustrating how they collectively shape the cancer genome, affect DNA repair mechanisms, and contribute to tumor evolution. However, the dynamic, context-dependent nature of epigenetic changes presents scientific and ethical challenges, particularly concerning privacy and clinical applicability. Focusing on lung cancer, we examine how specific epigenetic patterns function as biomarkers for distinguishing cancer subtypes and monitoring disease progression and relapse.

## 1. Introduction

In the complex and dynamic landscape of cancer biology, the interplay between genomic instability and epigenetic modifications has emerged as a major driver of tumor evolution. Among the various epigenetic changes, DNA methylation patterns and histone modification patterns can be thought of as “*Epigenetic Echoes*”—subtle yet persistent signals that reverberate through the genome, shaping cellular behavior and influencing disease outcomes. These echoes, often overlooked in the broader context of genetic mutations, provide crucial insights into the underlying mechanisms of cancer differentiation and progression. As cells accumulate genetic alterations due to genomic instability, these epigenetic marks can either amplify or mitigate the effects of these mutations, ultimately determining the trajectory of cancer development. Furthermore, the distinct methylation signatures left by these epigenetic echoes offer a powerful tool for distinguishing between cancer types and predicting relapse, paving the way for more precise diagnostics and personalized treatment strategies. This review delves into the intricate relationship between genomic instability and epigenetic modifications, exploring how these epigenetic echoes can be harnessed to improve an early cancer diagnosis, classification, and patient outcomes. First, we provide a concise overview of the individual aspects of genomic and epigenetic instability to give readers a foundation for understanding our main review focus on their interplay and its significance in lung cancer. Where appropriate, references to current reviews are included for readers seeking a more in-depth exploration of specific topics. These insights lay the groundwork for the final section, which explores future applications in lung cancer research, ethics, and personalized medicine.

### 1.1. Overview of Genomic Instability

#### 1.1.1. Mechanisms Leading to Genomic Instability

Imagine a typical cell in your body performing its essential functions: producing RNAs and proteins, maintaining cellular health, and carefully replicating its DNA. Now, consider a scenario where, due to an unexpected event, a mutation disrupts one of the critical proteins responsible for DNA maintenance and repair. This disruption initiates a cascade of events, leading to an accumulation of mutations and resulting in a state known as *genomic instability*, an unusually high frequency of DNA mutations and structural alterations that leaves the cell in a state of genomic chaos, with profound effects on cellular function and health [1].

*Genomic instability* can arise from multiple mechanisms, one of the primary being defects in genomic maintenance mechanisms, often called the “caretakers” of the genome. In normal cells, repair proteins, such as those encoded by BRCA1/2 and MLH1, function to fix DNA damage through mechanisms like homologous recombination (HR) and non-homologous end joining (NHEJ) for double-strand breaks, or DNA mismatch repair (MMR) for single-base errors [2]. When these repair systems are compromised, DNA errors accumulate, leading to further genomic instability and an increased likelihood of cancer development. Mutations and loss of heterozygosity (LOH) in tumor suppressor genes, such as TP53, compound this instability, removing critical safeguards that prevent uncontrolled cell growth [1,3]. Genomic alterations can range from *point mutations*, which alter single DNA bases, to *copy number alterations* (CNAs), involving large sections of the genome, while point mutations may have localized effects, CNAs can produce extensive shifts in gene expression and function, often with significant impact on cancer progression [4]. Chromosomal instability, including abnormalities in chromosome number (aneuploidy), also plays a significant role in cancer. Changes such as polyploidy, where chromosomes containing oncogenes are duplicated, can amplify cancer-driving properties. Conversely, nullisomies, losses of chromosomes containing tumor suppressor genes, reduce protective mechanisms against tumor formation [1]. These structural changes create a varied genomic architecture, depending on the concentration of oncogenes and tumor suppressor genes on specific chromosomal arms, adding complexity to the cellular landscape of cancer.

Other factors, such as replication stress and telomere dysfunction, further drive genomic instability. Telomeres, protective caps on chromosome ends, erode over time, and their loss leads to karyotypic instability and increased CNAs [5]. This is exacerbated by replication stress, particularly in regions called *common fragile sites*, which are prone to breaks and rearrangements during DNA replication. These fragile sites often experience damage in early cancer development, adding another layer to the instability that drives tumor progression [6].

#### 1.1.2. Consequences of Genomic Instability

Genomic instability has far-reaching consequences for cellular function, aging, and cancer development. As cells age, they accumulate mutations and chromosomal aberrations that weaken cellular function over time [7]. This process can be compared to a polluted river, where sediment and debris accumulate in the riverbed, mirroring the buildup of mutations within the genome. Additionally, the inflow of other river branches may be blocked, symbolizing disruptions to critical genetic pathways that are essential for maintaining cellular processes. As the river becomes polluted, the overall system deteriorates, just as genomic instability affects the cell’s ability to function properly. These disruptions distort or slow essential cellular processes, creating obstacles in genetic pathways and reducing cellular efficiency, much like a polluted river struggles to sustain its natural flow and ecological balance.

Mutations associated with genomic instability can activate or inactivate key cellular pathways that promote uncontrolled cell growth. For instance, activating mutations in oncogenes can hyperactivate growth factor pathways, making cells excessively responsive to signals that drive proliferation [8]. Conversely, inactivating mutations or loss of function (LOF) in tumor suppressor genes, such as TP53, impair the cell’s ability to detect DNA damage and induce apoptosis. This failure allows damaged cells to survive and proliferate, undermining essential feedback mechanisms that normally control growth. As a result, cells gain “*proliferative independence*”, enabling unchecked growth [8]. Some mutations and copy number alterations confer growth advantages, giving these cells a selective benefit over neighboring cells, which accelerates cancer progression.

Interestingly, genomic instability also grants a degree of *genomic plasticity*, allowing certain cells to adapt rapidly to environmental stressors. For instance, the presence of extrachromosomal DNA and mis-segregated chromosomes within micronuclei increases cellular diversity, contributing to cancer evolution and promoting tumor heterogeneity [1]. This plasticity is a double-edged sword for cancer cells, offering both an advantage and a risk. Tumors may exploit an optimal “*sweet spot*” of instability, achieving just enough mutations and rearrangements to drive rapid adaptation and resist treatment, without reaching levels so extreme that essential cellular functions collapse [9].

This balance allows tumors to evade many therapeutic strategies, complicating treatment efforts. Conversely, in cancers with extreme instability, the overwhelming genetic damage may lead to enhanced immunogenicity, potentially making them more detectable by the immune system than those with lower instability [9]. This can make cells more susceptible to treatment, as they struggle to survive under therapeutic pressure.

#### 1.1.3. Genomic Instability as a Cancer Hallmark

Genomic instability is a defining hallmark of cancer, underpinning the genetic diversity that enables tumors to grow, adapt, and resist treatment. Recognized as a critical feature in the influential “*hallmarks of cancer*” framework [8], genomic instability results from an increased mutation rate due to exposure to mutagenic agents or defects in genomic maintenance mechanisms. When these mechanisms fail, DNA accumulates mutations, leading to genomic instability that fuels cancer progression [8].

Ordinarily, *DNA damage* from uncontrolled cell growth (hyperproliferation) would trigger apoptosis, the programmed cell death pathway. However, cancer cells develop mechanisms to evade apoptosis, allowing them to survive and continue proliferating despite extensive DNA damage [8,10]. In particular, DNA damage in cancer cells, enforced by double-strand breaks, replication stress, and chromosomal mis-segregations, is not only a source of immediate genomic instability but can also be propagated through successive cell generations, amplifying instability and driving tumor progression. One major mechanism by which DNA damage is inherited is through chromosomal instability [11]. Mis-segregated chromosomes often form micronuclei, which are extranuclear bodies containing damaged DNA. Within micronuclei, the DNA is prone to aberrant replication and repair, leading to further chromosomal rearrangements and mutations. When these micronuclei reintegrate into the primary genome during subsequent cell cycles, they propagate the structural abnormalities to daughter cells. Experiments using live-cell imaging have demonstrated that micronuclei contribute to sustained aneuploidy and structural rearrangements, reinforcing the cycle of genomic instability in tumor cell populations [12]. In addition to the direct transmission of physical DNA damage, epigenetic changes at sites of damage also play an essential role in propagating instability. Following DNA damage, changes in DNA methylation patterns and histone modifications at break sites can alter gene expression, creating a “memory” of the damage that is inherited by daughter cells [13]. For instance, hypermethylation of DNA repair gene promoters (e.g., MGMT) or the persistence of repressive histone marks (e.g., H3K27me3) at damaged loci can compromise repair pathways in future generations, perpetuating genomic instability [14,15]. The findings by Naseli et al. underscore the importance of assessing DNA damage not only in adherent cells but also in detached cellular structures [16]. Their observations on fragmented metaphase chromosomes and inherited DNA damage support the notion that unresolved DNA breaks can be transmitted to daughter cells, amplifying instability. Interestingly, the hybrid instability observed in astrocytes reflects the nuanced interplay between damage and repair, aligning with the previously mentioned idea of a “sweet spot” of instability that allows cancer cells to thrive while avoiding complete genomic collapse.

These underlying genomic instabilities empower cancer cells to achieve hallmark traits, such as sustained proliferative signaling, evasion of growth suppressors, resistance to cell death, limitless replicative potential, angiogenesis, and metastatic ability. Furthermore, metabolic reprogramming [17] and immune evasion [18] have been recognized as additional hallmarks, underscoring a cancer’s adaptability in response to both genomic instability and environmental pressures. Different cancers exhibit unique patterns of mutations, and mutation profiles are often highly heterogeneous even within a single cancer type, for example in lung cancer, the leading cause of cancer-related deaths [19]. The processes contributing to genomic instability in lung cancer are spatially and temporally diverse, with different regions and stages of tumor development exhibiting distinct forms of instability [20]. This complexity shapes cancer evolution, making it a dynamic and challenging disease to treat. In lung cancer, genomic instability correlates with specific prognostic signatures that can indicate disease progression and inform treatment strategies [21,22]. Research using mathematical modeling has traced pathways of genomic instability in lung cancer, revealing key molecular targets that drive this instability and highlighting potential avenues for therapeutic intervention [22].

Genomic instability arises from failures in DNA maintenance mechanisms, leading to mutations and structural alterations that disrupt gene expression and accelerate tumor progression, while genomic instability enhances cancer adaptability and resistance to treatment, it also creates vulnerabilities that may be therapeutically targeted. Understanding these mechanisms is essential for developing strategies to disrupt cancer growth and evolution, highlighting genomic instability’s dual role as a disease driver and treatment opportunity.

### 1.2. Overview of Epigenetic Instability

**Figure 1:**
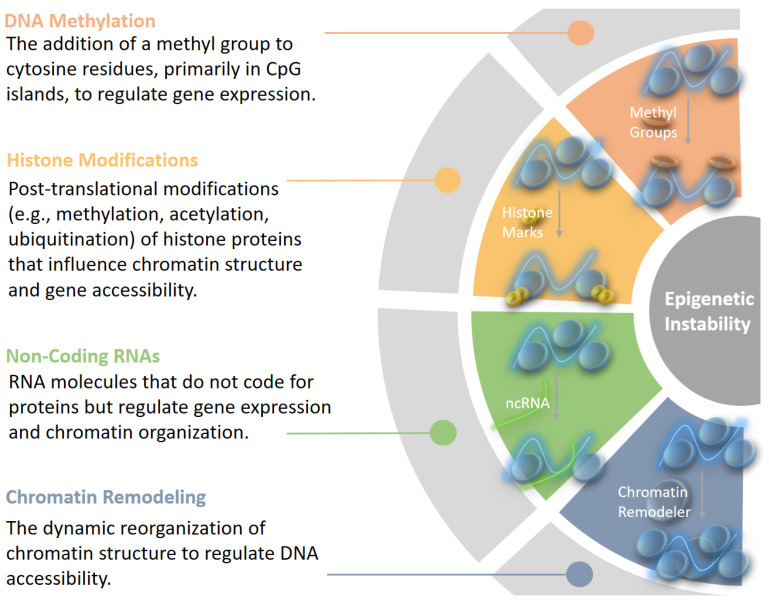
Conceptual illustration of Processes involved in *Epigenetic Instability.* Epigenetic instability refers to the disruption of the normal regulatory mechanisms that govern gene expression without altering the DNA sequence. These disruptions can lead to abnormal gene expression patterns and genomic instability, significantly contributing to tumor development and progression.

Epigenetic instability refers to changes in the epigenetic landscape of a cell that lead to alterations in gene expression without modifying the underlying DNA sequence. The epigenome can be envisioned as a dynamic canyon, with its structure shaping the echoes that influence genomic activity below. In normal cells, these epigenetic modifications are tightly controlled, maintaining genomic stability and balanced gene expression. However, when epigenetic regulation becomes unstable, it can lead to aberrant gene expression patterns, contributing to disease processes, particularly cancer [23]. Thus, understanding epigenetic instability is essential because it provides insights into how gene expression is misregulated in cancer, offering potential biomarkers for diagnosis and targets for therapeutic intervention (Figure 1). Epigenetic regulation involves several key mechanisms:

**DNA Methylation**: The addition of a methyl group to cytosine residues, particularly in CpG islands, is a well-studied mechanism for silencing gene expression. In cancer, DNA methylation patterns can be drastically altered, contributing to the repression of tumor suppressor genes or the activation of oncogenes. There are two primary types of methylation changes observed:

- **Global Hypomethylation**: This phenomenon involves the loss of methylation across the genome, which can lead to chromosomal instability and activate oncogenes. It is commonly observed in various cancers.
- **Localized Hypermethylation**: Specific regions, particularly the promoters of tumor suppressor genes, can undergo hypermethylation, resulting in gene silencing. This silencing removes critical checks on cell growth and division, thus enabling tumor growth.

**Histone Modifications**: Histones, the protein components around which DNA winds, can undergo post-translational modifications, such as methylation, acetylation, and ubiquitination. These modifications influence chromatin structure, thereby regulating gene accessibility and transcriptional activity. For instance, the mono-methylation of H2BK120 has been shown to prevent ubiquitination of this histone, stabilizing it in a way that downregulates the transcription of tumor-suppressor genes [24]. Specific patterns of histone modifications are associated with cancer, with certain modifications being linked to oncogene activation and tumor suppressor gene silencing [25]. Histone acetylation, for instance, is typically associated with open chromatin and active transcription. In cancer, there can be a loss of acetylation at tumor suppressor genes and increased acetylation at oncogenes, contributing to abnormal cell growth [26,27]. Recent work has highlighted non-mutational epigenetic reprogramming as a *hallmark of cancer* [28], where cancer cells undergo broad, non-genetic shifts in their epigenetic landscape, creating an environment that supports unchecked cell proliferation and survival.

**Non-coding RNAs (ncRNAs)**: Non-coding RNAs, including microRNAs (miRNAs), long non-coding RNAs (lncRNAs), and PIWI-interacting RNAs (piRNAs), are known to contribute to gene regulation and genomic stability [29]. They interact with DNA methylation and histone modification machinery, contributing to fine-tuning gene expression and the organization of chromatin. In cancer, specific miRNAs are known to silence tumor suppressor genes or activate oncogenes in cancer, while lncRNAs can recruit epigenetic complexes to specific genomic loci, altering the local epigenetic state [30]. In addition, piRNAs are a type of small non-coding RNA, typically 24–31 nucleotides in length, that interact with PIWI proteins, a subfamily of Argonaute proteins [31]. They also have the functionality in maintaining genomic stability by silencing transposable elements and regulating epigenetic states. When their pathways are disrupted, the resulting transposon reactivation and epigenetic dysregulation can contribute to genomic instability or act as oncogenic or tumor-suppressive factors [32]. Furthermore, recent studies have shown that methylated RNAs play a significant role in cancer, influencing processes like RNA stability, 3D structure, splicing, and translation efficiency [24]. These modifications add an additional layer to the epigenetic regulation in cancer, with far-reaching effects on gene expression and cellular behavior. Methylated RNAs, in particular, present a fascinating area for further research, as they offer insights into both the stability of the transcriptome and the post-transcriptional regulation of gene expression in cancer [33,34].

**Chromatin remodeling**: As an essential process for regulating DNA accessibility, chromatin remodeling plays a major role in maintaining genomic and epigenetic stability [35]. It involves ATP-dependent complexes (e.g., SWI/SNF) that reorganize nucleosomes and interact with histone modifiers to control chromatin states. Dysregulation of chromatin remodeling contributes to epigenetic instability through several mechanisms:

- **Disrupted Chromatin Accessibility**: Aberrant remodeling can result in either hyper-compacted or excessively open chromatin, impairing transcription and DNA repair processes [36].
- **Histone Modification Alterations**: Mutations in remodelers (e.g., SWI/SNF components) disrupt interactions with histone-modifying enzymes, silencing tumor suppressor genes or activating oncogenes [37].
- **Nucleosome Instability**: Improper incorporation of histone variants destabilizes chromatin, increasing susceptibility to DNA damage [38].
- **Compromised DNA Repair**: Defective remodeling hinders repair pathways like homologous recombination, leading to the accumulation of mutations and further epigenetic changes [39].

Such dysregulation fosters tumorigenesis by enabling genomic instability, tumor heterogeneity, and therapy resistance [35].

This overview highlights the complex role of epigenetic instability in cancer, where disruptions in DNA methylation, histone modifications, non-coding RNAs, and chromatin remodeling collectively disturb gene regulation. These epigenetic alterations not only drive tumor progression but also intertwine with genomic instability, creating a feedback loop that exacerbates genetic mutations and chromosomal alterations.

## 2. Relationship Between Genomic and Epigenetic Instability

**Figure 2:**
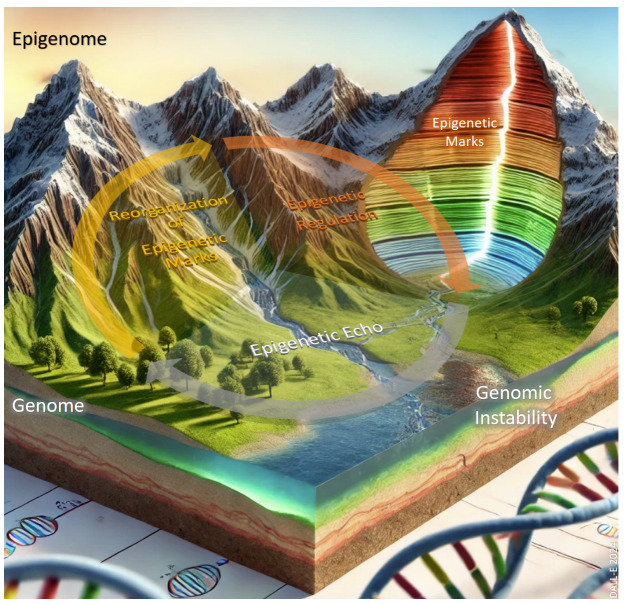
Conceptual illustration of *epigenetic echoes*, persistent and dynamic changes in the epigenome that influence the genome and genomic (in)stability. The mountains symbolize the epigenome, with layered formations representing epigenetic marks, such as DNA methylation and histone modifications. Orange and yellow arrows indicate processes like ’Reorganization of Epigenetic Marks’ and ’Epigenetic Regulation’, which cascade down into the valley, representing the genome. These echoes impact the genomic landscape, including regions of genomic instability (pictured as a polluted river), affecting DNA repair and overall genomic integrity. The image highlights the interplay between epigenetic regulation and genomic stability, illustrating how epigenetic changes can either reinforce or disrupt genome function. Background generated by DALL-E 2024; conceptual design, content, and annotations of the figure were entirely created by the authors.

*Epigenetic echoes* are persistent changes in the epigenome represented as epigenetic marks, such as DNA methylation, histone modifications, and non-coding RNA regulation, that reflect and amplify underlying genomic instability. Again, imagine the epigenome as a canyon and the genome as the valley it surrounds. The echoes reverberating through the canyon are shaped by its structure, its walls, depth, and terrain, much like how the epigenetic marks reflect and respond to genomic mutations and structural changes (Figure 2). Over time, as the epigenome (the canyon) changes, it can alter the accessibility of the genome (the valley), either exposing parts of the genome to greater activity or shielding regions critical for DNA repair and stability. If these critical regions are disrupted, with mutations accumulating over time, the smooth flow of the river in the valley becomes polluted with debris, symbolizing genomic instability. As a result, the entire (eco)system risks losing control, emphasizing the dynamic interplay between epigenome and genome in maintaining cellular health.

The interplay between genomic and epigenomic instability adds layers of complexity to cancer evolution, influencing tumor heterogeneity, resistance to treatment, and the potential for relapse. Understanding how these interconnected processes unfold is essential for accurately distinguishing cancer subtypes and predicting clinical outcomes, paving the way for more refined, early-stage diagnostics and therapeutic strategies.

### 2.1. Epigenetic Crosstalk Between Mountains and Valleys

Epigenetic and genomic instability are thus *indirectly* connected through *echoed* intricate mechanisms, such as the crosstalk between DNA methylation and histone modifications. For instance, 5-methylcytosine (5mC), a common DNA methylation modification, can impact DNA accessibility, creating a more compact chromatin structure that represses gene expression [40]. This compact chromatin state reduces access to transcriptional machinery, silencing genes, including those involved in DNA repair. Additionally, altered DNA methylation can promote the formation of non-canonical DNA structures like G-quadruplexes, which destabilize chromatin and create regions susceptible to further genomic damage [41,42]. These structures contribute to genomic instability by introducing potential sites for mutations and rearrangements, which can further alter epigenetic patterns in a vicious cycle.

Non-coding RNAs add another layer to this complex interplay by interacting directly with chromatin remodeling processes to regulate gene expression. For example, lncRNAs, such as HOTAIR recruit chromatin modifiers like Polycomb Repressive Complex 2 (PRC2) to specific genomic loci, establishing repressive marks such as H3K27me3 that silence tumor suppressor genes [43]. Similarly, miRNAs influence chromatin accessibility by regulating the expression of enzymes like histone deacetylases (HDACs) and DNA methyltransferases (DNMTs). Dysregulation of these ncRNAs can lead to widespread epigenetic changes that exacerbate genomic instability, particularly in cancer cells [44]. Circular RNAs (circRNAs) also contribute by acting as “sponges” for miRNAs, preventing them from regulating chromatin-associated enzymes [45]. This indirect regulation can amplify epigenetic instability by allowing unchecked chromatin remodeling activity. For instance, circRNAs in lung cancer have been implicated in modulating the activity of miRNAs that target key chromatin remodeling proteins, further contributing to the epigenomic chaos characteristic of tumor progression [46].

Genomic instability, including mutations and chromosomal rearrangements, can disrupt normal methylation patterns, leading to widespread epigenetic alterations. For instance, structural alterations in the genome can interfere with the distribution of DNA methylation and histone modification marks, impacting gene regulation across large chromosomal regions [47]. Thus, feedback loops between genomic and epigenetic instability also emerge as the genome ages, where accumulated DNA damage and mutations progressively lead to dysregulated epigenetic patterns [7]. This is particularly relevant in cancer, where genomic and epigenetic changes co-evolve, contributing to the adaptability and survival of cancer cells under selective pressures, such as therapy. Telomere shortening is another common cause of genomic instability in aging and cancer cells [48]. Short or dysfunctional telomeres can trigger epigenetic changes in nearby chromatin, leading to increased DNA methylation and histone modifications that further suppress genomic stability. This connection between telomere dysfunction and epigenetic alteration is particularly relevant in tumor cells with a high replication rate, as it drives both genomic and epigenetic dysregulation [49]. Non-coding RNAs also influence telomere maintenance by regulating the expression of shelterin proteins and telomerase components, highlighting their multifaceted roles in maintaining genomic integrity [50,51]. Targeting the epigenetic modifications associated with telomere instability could offer potential therapeutic strategies for certain cancer types.

### 2.2. Epigenetic Regulation of DNA Repair Genes

Epigenetic changes play a critical role in regulating DNA repair pathways, a key mechanism linking epigenetic instability to genomic instability. For instance, methylation-induced silencing of DNA repair genes [52] compromises a cell’s ability to correct DNA damage, thereby accelerating mutation rates. Key enzymes involved in epigenetic regulation can also drive genomic instability. EZH2, a histone methyltransferase, has been shown to trigger genomic instability and activate oncogenic signaling in breast tumor-initiating cells, a subset of cancer stem cells implicated in breast cancer progression [53]. EZH2 is now recognized as a major epigenetic regulator in the tumor microenvironment and a promising target in immunotherapy [54].

Chromatin remodeling complexes, such as SWI/SNF, INO80, and CHD, have dual roles in regulating chromatin accessibility and in coordinating DNA repair processes [55]. These complexes can modulate both genome and epigenome stability by facilitating or inhibiting the accessibility of repair proteins to damaged DNA [56]. Mutations in chromatin-remodeling genes, common in various cancers, disrupt DNA repair pathways and contribute to epigenetic instability. This connection can drive genome-wide instability and create vulnerabilities that could be targeted therapeutically, as some cancer cells become highly reliant on alternative repair pathways.

Replication stress is a form of genomic instability that results from errors during DNA replication, leading to DNA damage and mutations [57]. Interestingly, replication stress can disrupt normal DNA methylation patterns, particularly in regions with high rates of CpG sites, affecting gene regulation on a broader scale. This interaction is significant in cancer, where replication stress can lead to localized DNA demethylation or abnormal methylation, which may silence or activate genes enhancing tumor development [58]. Exploring this interplay could provide insight into how replication stress-induced epigenetic alterations contribute to tumor heterogeneity and the evolution of cancer subtypes.

Epigenetic alterations induced by therapy can lead to treatment resistance. DNA methylation patterns in tumor cells often change following radiotherapy or chemotherapy, with some of these changes promoting resistance [59]. Such DNA methylation alterations can serve as predictive biomarkers, indicating when therapies need to be adjusted. Mechanisms behind resistance can be multifactorial, stemming from genetic mutations and epigenetic reprogramming, hypoxia, and shifts in the tumor microenvironment. Understanding these mechanisms is crucial for developing strategies to counteract resistance, re-sensitizing tumors to treatment and improving patient outcomes [60].

### 2.3. Implications for Tumor Evolution and Heterogeneity

The interaction between genomic and epigenetic instability fosters the development of diverse tumor cell populations, a phenomenon critical to tumor evolution and progression. Recent studies, such as those by Terekhanova et al. [61], have employed single-nucleus chromatin accessibility data across multiple cancer types to reveal distinct epigenetic drivers associated with cancer transitions. Such findings illustrate the role of epigenetic regulation during cancer progression, highlighting how this instability contributes to cellular heterogeneity and plasticity. Moreover, a pan-cancer atlas integrating chromatin accessibility and transcriptomic data from over a million cells has identified epigenetic factors driving tumor evolution and subtype-specific transitions [61]. These findings reinforce the importance of considering epigenetic dynamics alongside genomic changes in understanding cancer complexity.

Epigenetic reprogramming without underlying genetic mutations, termed non-mutational epigenetic reprogramming, is emerging as an additional hallmark of cancer [28,62,63]. In certain cancers, such as small-cell and non-small-cell lung cancer (SCLC and NSCLC), lineage reprogramming can lead to dynamic, reversible shifts between cancer subtypes [64]. This plasticity enables cancer cells to adapt to environmental pressures, including therapies, resulting in diverse cell populations that complicate treatment. Studies show that epigenomic state transitions characterize tumor progression, with cancer cells transitioning through various epigenetic states during disease progression [65]. These state changes allow tumors to exploit lineage plasticity, fueling adaptability and increasing the potential for relapse.

Hypomethylation of retrotransposons, such as LINE-1 elements, is correlated with genomic instability in cancers, particularly in non-small-cell lung cancer [66]. LINE-1 retrotransposition, a process that leads to gene disruption and increased mutation rates, further contributes to tumor development. Retrotransposons not only destabilize the genome but also impact metabolic reprogramming and have been proposed as prognostic markers in lung cancer [67].

Sirtuin (SIRT) genes, a family of the oxidized form of Nicotinamide Adenine Dinucleotide (NAD+)-dependent deacetylases, play a pivotal role in epigenetic modulation and genomic stability [68]. By deacetylating histones, such as H3 and H4, sirtuins regulate chromatin structure and transcriptional activity, contributing to the silencing of tumor suppressor genes or the activation of DNA repair pathways. Beyond histones, sirtuins also target non-histone proteins, such as p53, influencing apoptosis, senescence, and genomic integrity [69]. SIRT1 and SIRT6, in particular, enhance DNA repair mechanisms like base excision repair and homologous recombination, while also stabilizing telomeres and mitigating replication stress [70,71]. These activities position sirtuins at the intersection of genomic and epigenomic instability, amplifying or mitigating epigenetic echoes that propagate through the genome. Notably, sirtuins exhibit a dual role in cancer, acting as tumor suppressors by preserving genomic stability but also promoting oncogenesis when dysregulated [72]. Their involvement underscores their potential as both biomarkers for cancer progression and therapeutic targets for restoring epigenetic balance, making them a critical component of the dynamic interplay between the epigenome and genome.

The intricate relationship between genomic instability and epigenetic alterations unveils an increasingly complex network that underpins cancer development, cellular diversity, and therapy resistance. This interplay highlights the need to look beyond DNA sequence mutations to fully understand the mechanisms driving tumor progression and adaptability. As cancer research continues to evolve, integrating genomic and epigenomic insights becomes essential for identifying robust biomarkers and therapeutic targets at early stages.

## 3. Epigenetic Biomarkers for Lung Cancer Type Distinction and Relapse

Epigenetic modifications can silence tumor suppressor genes, activate oncogenes, or create new vulnerabilities that further propagate genomic instability. This dynamic interplay drives cancer progression and contributes to tumor heterogeneity. In lung cancer, for instance, epigenetic biomarkers, such as DNA methylation patterns play prominent roles in distinguishing subtypes and monitoring disease progression. Leveraging advanced technologies and integrative genomic and epigenomic analyses, these biomarkers are becoming invaluable for diagnostics and prognostics, offering more precise and non-invasive tools for managing cancer [73]. Table 1 summarizes key epigenetic markers implicated in early lung cancer detection. These include DNA methylation markers, such as RASSF1A and CDKN2A, non-coding RNA regulators, like miR-21 and MALAT1 [73], and emerging diagnostic tools based on histone modifications and liquid biopsy technologies.

### 3.1. Biomarker Identification via Epigenetic Marks

Epigenetic marks, primarily DNA methylation, serve as stable and tissue-specific biomarkers that can distinguish between cancer types and subtypes. Technologies for methylation analysis have evolved, from initial methods focused on quantifying 5-methyl-cytosine to more advanced platforms that map DNA methylation across the entire genome at single-nucleotide resolution [89]. These platforms support the creation of high-resolution methylation maps, essential for accurate cancer classification and monitoring. Advances in next-generation sequencing (NGS) and microarray technology have allowed high-resolution mapping of these marks, uncovering specific methylation signatures associated with various cancer types [90]. This capability has expanded to include single-cell and low-input technologies, enabling analysis of cell-free DNA (cfDNA) in liquid biopsies, which offers a non-invasive means to detect and monitor cancer [91].

**Epigenetic Biomarkers in Lung Cancer Subtypes**: Recent studies demonstrate that distinct DNA methylation profiles exist for different lung cancer subtypes, providing a foundation for molecular classification that can guide clinical decision-making [92,93,94].

Epigenetic profiles have the potential to differentiate among lung cancer subtypes, such as SCLC and NSCLC. For example, a multi-omics analysis by Yang et al. [95] integrated mRNA expression and methylation patterns associated with overall survival, identifying prognostic molecular subtypes for early-stage NSCLC. Similarly, promoter hypermethylation of genes involved in cell cycle regulation, DNA damage response, and immune modulation were found to be associated with prognosis in NSCLC [96,97].

**Epigenetic Indicators for Early Detection of Lung Cancer**: Hypermethylation of genes like RARB, CDKN2A, and MGMT have emerged as promising biomarkers for lung cancer early detection, prognosis, and prediction of therapeutic response [98]. RARB (Retinoic Acid Receptor Beta) plays a critical role in cell differentiation and growth inhibition, while CDKN2A (Cyclin-Dependent Kinase Inhibitor 2A) is a tumor suppressor involved in cell cycle regulation. MGMT (O6-Methylguanine-DNA Methyltransferase) is essential for DNA repair, specifically in reversing alkylation damage. Methylation patterns of these genes have been shown to vary across NSCLC subtypes and stages, highlighting their potential utility for differential diagnosis, disease monitoring, and stratifying patients for personalized therapeutic approaches [99,100].

### 3.2. Practical Classification and Screening Applications in Oncology

DNA methylome analysis has emerged as a powerful tool for molecular tumor classification in non-invasive diagnostics. Sill et al. [101] highlighted that genome-wide DNA methylation profiling impacts diagnostic workflows and patient stratification, especially in brain tumors and increasingly in other cancers like sarcomas and hematologic malignancies. In lung cancer, cfDNA methylation profiles allow for the classification of tumor types from a simple blood sample, minimizing the need for invasive procedures.

As part of early detection strategies, researchers are evaluating if methylation-based liquid biopsies can detect cancer months or even years before current clinical methods, with the goal of identifying cancers at an earlier, more curable stage [102]. However, one of the primary challenges in cfDNA-based liquid biopsies is multi-cancer early detection, where the goal is to accurately diagnose cancer type from cfDNA in asymptomatic individuals. Thus, emerging methodologies interpret cancer-type-specific genomic and epigenomic features, offering potential breakthroughs in cfDNA-based diagnostics at early cancer stages [103].

For Methylation-Based Pan-Cancer Screening, a key opportunity lies in the development of circulating tumor DNA (ctDNA) methylation assays for multi-cancer early detection (stMCED), which screen for multiple cancer types with a single test. This strategy leverages fixed specificity and aggregated disease prevalence to improve positive predictive value, potentially enhancing population health outcomes through early and minimally invasive diagnostics [104]. Studies, such as Zhang et al. [105], illustrate the power of methylation-based approaches across multiple cancers. By combining advanced computational techniques, the authors were able to distinguish 33 tumor types through methylation-based decision rules. This demonstrates the broad applicability of methylation biomarkers in cancer diagnosis and the potential for identifying novel therapeutic screening approaches.

### 3.3. Epigenetic Indicators of Cancer Relapse

Epigenetic biomarkers are also emerging as valuable tools for monitoring cancer relapse due to their ability to reveal persistent, dynamic changes that signal disease recurrence. These biomarkers, particularly DNA methylation and histone modifications, can indicate residual disease post-treatment, help in assessing the risk of relapse, and support personalized treatment strategies [106]. Given the reversible nature of epigenetic changes, they are also promising therapeutic targets for managing relapse in lung cancer patients. For instance, the reappearance of the H3K27me3 mark in certain cancers has been linked to relapse, as observed in studies where eliminating H3K27me3 with histone methyltransferase inhibitors showed clinical benefit and reprogrammed the cancer epigenome [107]. These findings underscore the predictive power of specific epigenetic changes and their potential for guiding clinical interventions aimed at preventing relapse.

**Persistent Epigenetic Changes in Relapse Prediction and Therapy**: After initial treatment, specific methylation patterns can persist in residual cancer cells and serve as indicators of potential relapse. The persistence of these modifications, even in the absence of visible tumor burden, can reveal subtle changes that precede clinical relapse. Given the reversible nature of many epigenetic changes, therapies targeting epigenetic modifiers offer the potential to restore the cancer epigenome to a more stable state, either as a monotherapy or combined with other treatments, including immunotherapies [23,108]. By targeting these persistent methylation marks, therapeutic interventions may delay or prevent relapse in patients at high risk. In hematologic malignancies, abnormal DNA methylation patterns, including genome-wide hypomethylation and hypermethylation or hypomethylation of CpG islands, are frequently observed and have proven valuable as relapse indicators [109]. In lung cancer, particularly NSCLC, histone modifications play a significant role in tumor progression. Studies have shown that decreased levels of H3K4me3, a histone modification associated with gene activation, correlate with poor prognosis and greater invasiveness in NSCLC. Similarly, alterations in histone acetylation have been linked to tumor aggressiveness and treatment resistance [110]. Monitoring these histone modifications post-treatment can help predict relapse and guide subsequent therapeutic strategies.

**Epigenetic Dynamics and Multi-Omics Integration in Relapse Prediction**: Cancer heterogeneity and resistance to therapy are partly driven by dynamic and reversible epigenetic modifications that allow cancer cells to adapt under selective pressures. Epigenetic modifications contribute to diverse cell populations within the same tumor, enabling them to survive treatments that target only specific cell types [111]. However, understanding these mechanisms remains challenging due to the complexity and variability of the epigenetic landscape in cancer, which is intertwined with genetic alterations. Integrating epigenetic markers with other omics layers, such as transcriptomics, proteomics, and metabolomics, can provide a comprehensive approach for relapse prediction. Multi-omics integration, especially through minimally invasive liquid biopsies, allows for repeated, non-invasive monitoring of relapse indicators and offers a robust approach to track treatment response and resistance in real time [112]. Single-cell and multi-omics technologies have revolutionized our ability to trace cell lineages, identify tissue- and cell-specific patterns, and map the spatial architecture of tumors. These advancements contribute to a deeper understanding of tumor immunology and cancer genetics, essential for identifying precise biomarkers for relapse prediction [113].

Epigenetic biomarkers, including DNA methylation and histone modification patterns, offer essential insights into lung cancer diagnosis and relapse by allowing early detection of residual disease, predicting treatment response, and enabling personalized patient care. The integration of epigenetic data with other omics and advanced single-cell technologies is making comprehensive relapse prediction and prevention increasingly attainable.

## 4. Advancing Therapeutic Strategies—Next Steps and Unmet Needs

Epigenetics and genomic instability offer exciting opportunities for cancer therapy, particularly in personalized and targeted treatment approaches. However, significant challenges remain in optimizing treatment efficacy, managing resistance, and addressing ethical considerations. Integrating multi-omics data is crucial to developing a comprehensive understanding of cancer behavior and identifying new therapeutic vulnerabilities.

### 4.1. Targeting Genomic Instability

Tumors with moderate genomic instability often develop resistance to therapies, as they acquire the ability to survive under a variety of stress conditions. However, tumors with excessive genomic rearrangement may be more vulnerable to targeted treatments, creating a therapeutic “sweet spot” for treating genomically unstable cancers [9]. In cancers with Homologous Recombination Deficiency (HRD), such as advanced chordomas, targeting the deficiency in DNA repair can be particularly effective [114]. HRD-targeted therapies, such as PARP inhibitors, exploit the inability of these tumors to repair DNA damage, leading to increased cell death. This approach underscores the importance of tailoring treatments based on the specific genomic instability profile of each tumor.

### 4.2. Targeting Epigenetic Modifications

**Figure 3:**
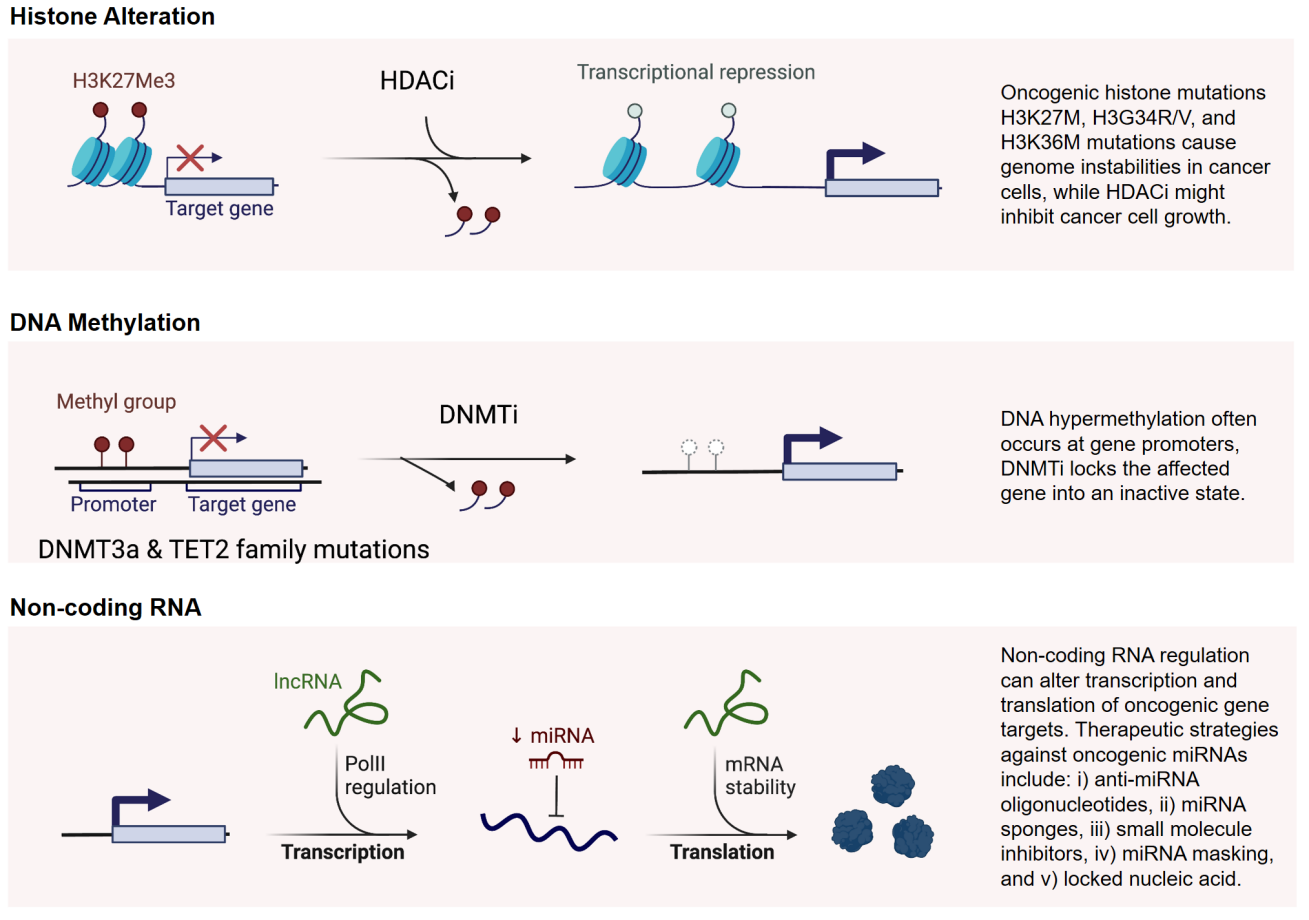
Epigenetic Alterations in Cancer and Treatment. The figure illustrates key epigenetic mechanisms involved in cancer progression and their corresponding therapeutic interventions, i.e., Histone Modifications, DNA Methylation, and Non-Coding RNAs. This representation highlights the potential of epigenetic therapies in targeting cancer-specific alterations.

The development of drugs targeting epigenetic enzymes, such as histone methyltransferases and DNA methyltransferases, has expanded therapeutic possibilities in cancer. These inhibitors can potentially reverse abnormal epigenetic states, restoring normal gene expression patterns and increasing treatment sensitivity [115]. For example, inhibitors targeting EZH2, a histone methyltransferase, are under investigation for treating cancers with specific epigenetic alterations [116]. Here, targeting chromatin remodeling pathways through Tazemetostat (an EZH2 inhibitor) in combination with other drugs have shown efficacy in certain specific lung cancer subtypes [117]. In addition to histone methyltransferase inhibitors, histone deacetylase inhibitors (HDACi) and DNA methyltransferase inhibitors (DNMTi) have gained increased attention in epigenetic therapy for lung cancer (Figure 3).

**Histone Deacetylase Inhibitors**: In particular, HDAC inhibitors work by blocking histone deacetylases, enzymes that remove acetyl groups from histones, leading to chromatin compaction and transcriptional silencing [118]. By inhibiting HDAC activity, these drugs induce hyperacetylation of histones, resulting in chromatin relaxation and reactivation of silenced tumor suppressor genes (Figure 3). In lung cancer, HDAC inhibitors like vorinostat and romidepsin have shown promise in restoring tumor suppressor activity, sensitizing cancer cells to chemotherapy and immunotherapy, and enhancing anti-tumor immune responses [119]. However, challenges such as off-target toxicities and the need for predictive biomarkers remain significant hurdles [120].

**DNA Methyltransferase Inhibitors**: DNMT inhibitors, such as azacitidine and decitabine, target the hypermethylation of tumor suppressor gene promoters, a hallmark of many cancers, including lung cancer [121]. These agents incorporate into DNA and irreversibly bind to DNA methyltransferases, leading to passive demethylation during DNA replication (Figure 3). This process reactivates genes involved in apoptosis, cell cycle control, and immune modulation. Preclinical and clinical studies have shown that DNMTi can enhance immune recognition of cancer cells and synergize with immune checkpoint inhibitors, offering new avenues for combination therapies in NSCLC [122]. Similar to HDACi, DNMTi face challenges, such as off-target effects, limited efficacy as monotherapies, and the need for robust biomarkers to guide patient selection.

The combination of DNMTi with other epigenetic drugs, such as HDACi, also holds great promise. This dual inhibition strategy can synergistically reactivate silenced genes and modulate the tumor microenvironment, addressing tumor heterogeneity and therapy resistance [123,124].

In addition, SIRTs are also gaining recognition as versatile therapeutic targets, given their critical roles in modulating epigenetic modifications, enhancing DNA repair, regulating cellular metabolism, and managing stress responses, making them valuable for novel lung cancer treatment strategies [125,126,127]. Despite these advancements, applying epigenetic therapies in clinical settings poses challenges. The reversible and dynamic nature of epigenetic changes requires careful consideration of dosage and timing to avoid unintended effects. In breast cancer, for example, one challenge is the potential for epigenetic modifications to impact cells beyond the tumor, highlighting the need for therapies with high specificity to minimize off-target effects [128]. Since the cancer epigenome plays a significant role in how cells respond to treatment, a personalized approach is essential. Tailoring epigenetic therapies based on each patient’s unique epigenetic landscape, and maybe reshape its marks, offers the potential to maximize therapeutic benefit while minimizing adverse effects, aligning with the goals of precision medicine.

Table 2 summarizes active clinical trials investigating epigenetic therapies in lung cancer, highlighting their mechanisms, trial phases, and therapeutic focus. These trials emphasize the potential of epigenetic drugs in combination with immune checkpoint inhibitors and as standalone treatments for specific lung cancer subtypes.

### 4.3. Natural Epi-Drugs as Emerging Tools for Epigenetic Modulation

Recent advancements in epigenetic research have highlighted the therapeutic potential of natural compounds, often termed natural epi-drugs, for modulating epigenetic pathways [129]. These compounds, derived from plants, dietary sources, or microbial metabolites, have demonstrated the ability to regulate DNA methylation, histone modifications, and non-coding RNA expression. Their broad-spectrum activity, low toxicity profiles, and ability to target multiple epigenetic regulators make them promising candidates for cancer therapy [130]. Natural epi-drugs exert their effects through a variety of mechanisms:

**DNA Methylation Modulation**: Compounds such as epigallocatechin gallate (EGCG), a polyphenol found in green tea, and genistein, an isoflavone from soy, have been shown to inhibit DNMTs as well [131]. This inhibition leads to the demethylation of tumor suppressor gene promoters, reactivating their expression and suppressing tumor progression.

**Histone Modifications**: Natural compounds like curcumin (from turmeric) and resveratrol (from grapes and red wine) can regulate histone acetylation and methylation [132]. For instance, curcumin inhibits histone acetyltransferases (HATs), thereby restoring a balanced chromatin state that favors tumor suppressor gene expression. Resveratrol has also been shown to affect histone deacetylases (HDACs), promoting chromatin relaxation and reactivating silenced genes.

**Non-Coding RNA Regulation**: Natural epi-drugs can influence the expression of microRNAs (miRNAs) and long non-coding RNAs (lncRNAs). For example, EGCG has been shown to modulate miRNA expression, targeting oncogenic pathways and enhancing tumor suppressive mechanisms [133].

Natural epi-drugs, as highlighted in recent studies by Volpes et al., represent a promising avenue for targeting the dynamic and reversible nature of epigenetic changes in cancer, including lung cancer [134]. Their ability to modulate epigenetic landscapes opens new possibilities for therapies [135].

### 4.4. Ethics of Implementing Epigenomics Technologies in Cancer Screening and Treatment

Epigenomic screening technologies show promise in early cancer detection, offering the potential for preventive measures and timely intervention. The ethical considerations surrounding epigenetics differ from those of other genomics data in some important ways, though they share commonalities as well. Epigenetics introduces unique ethical challenges primarily due to the dynamic and environmental responsiveness of epigenetic marks, which are not static like genomic sequences [136]. There is a growing need to address ethical considerations surrounding the use of epigenetic data for early disease detection. A scoping review identified gaps in research on ethical aspects of early detection, including concerns about overestimating health capacity and preventing undue blame on individuals for their disease risk [137]. Epigenetics captures not only genetic predispositions but also modifications that reflect lifestyle choices, environmental exposures, and even socio-economic conditions [138]. This responsiveness raises specific ethical questions about privacy and potential stigma. For instance, if an epigenetic profile indicates a high cancer risk due to environmental exposures, individuals may feel blamed for factors outside their control, or employers and insurers may misuse such information. Conversely, genomic data largely represents inherent traits that are stable and less modifiable by lifestyle or environment, while genomic data also has privacy implications, it lacks the sensitivity of epigenetic data in reflecting an individual’s lived experiences. Earlier detection is indeed a critical point in both genomics and epigenetics, as both fields aim to identify cancer risks before symptoms arise. However, epigenetics may offer insights into very early or even pre-cancerous stages of disease by detecting environmental or lifestyle-induced changes. This capacity for early intervention is promising but raises questions about how individuals should be informed and whether public health systems are equipped to handle widespread early screening. With genomic data, early detection focuses primarily on identifying inherited cancer risks, such as BRCA1/2 mutations, where decisions are based on genetic predispositions rather than potentially modifiable factors. As epigenomics technologies advance, developing ethical frameworks will be critical to ensuring responsible use in public health.

### 4.5. Future Directions—Multi-Omics Integration for Comprehensive Cancer Therapy?

Integrating multi-omics data, such as genomics, transcriptomics, and epigenomics, offers a more holistic view of cancer behavior and enables identification of new therapeutic targets. Athieniti et al. [139] observed that omics combinations are often chosen based on biological evidence and the specific objectives of a study, with transcriptomics and epigenomics particularly valuable for understanding regulatory processes, while proteomics and metabolomics are closer to phenotypic insights relevant to diagnostics. The integration of epigenomic, transcriptomic, proteomic, and metabolomic data in large cohorts, such as The Cancer Genome Atlas (TCGA), has provided valuable insights into cancer phenotypes and systemic dysregulation associated with specific cancer types. These integrative analyses are essential for developing precise, multi-layered therapeutic strategies and advancing our understanding of cancer’s complexity [140,141,142]. An interesting computational aspect brings LungDWM, which is a model that leverages attention-based feature encoding and generative adversarial learning to integrate and impute missing omics data, enabling accurate subtype diagnosis and interpretability in lung cancer [143]. Such models highlight the diagnostic potential of multi-omics data integration and represent a promising tool for translating omics data into actionable insights for treatment planning.

Integrating epigenomics with other omics layers (e.g., genomics, proteomics) could reveal new therapeutic vulnerabilities in cancers with high epigenetic instability. Studies have shown that linking genomics with DNA methylation and other omics layers can improve our understanding of drug resistance mechanisms and provide mechanistic insights into cancer progression [144]. A potential downside of multi-omics integration for comprehensive cancer therapy, particularly when incorporating epigenomics data, is the complexity of data interpretation and analysis. Epigenomics data are highly context-dependent, reflecting not only inherent genetic predispositions but also dynamic environmental and lifestyle influences. Unlike static genomic data, epigenomic information varies across tissues, time points, and even in response to environmental changes [145]. This variability complicates interpretation and may reduce the reproducibility and consistency of findings across patients. Clinicians may find it challenging to translate these nuanced epigenomic signatures into clear, actionable therapeutic decisions, especially in the context of dynamic disease states like cancer.

The therapeutic potential of targeting epigenetic and genomic instability in cancer is vast but requires further research and refinement. Advancing personalized medicine approaches, multi-omics integration, and ethical frameworks will be essential to realizing the full potential of these therapies. As the field progresses, a comprehensive approach that combines genomic, epigenomic, and other omics data will enable more precise targeting of cancer vulnerabilities.

## 5. Conclusions

In summary, the intricate relationship between genomic and epigenomic instability plays a crucial role in cancer progression, heterogeneity, and resistance to treatment. Epigenetic modifications, such as DNA methylation and histone modifications, act as epigenetic echoes, persistent yet adaptable markers that reflect the underlying genetic chaos driving cancer. These modifications serve as valuable biomarkers for distinguishing cancer subtypes and predicting relapse while also offering promising therapeutic targets. Advances in multi-omics integration are paving the way for a more comprehensive understanding of cancer, uncovering new vulnerabilities within these genomic echoes that can be exploited for treatment.

Clinically, these insights hold significant potential for the development of personalized medicine, where treatments are tailored to each patient’s unique genomic and epigenomic profile. The integration of epigenetic biomarkers into non-invasive diagnostic tools, such as liquid biopsies, is particularly promising, enabling earlier detection, real-time monitoring, and adaptive treatment strategies that could improve patient outcomes. However, the dynamic and context-dependent nature of epigenetic data also calls for ethical frameworks that address privacy, accessibility, and responsible data use in clinical settings.

Future research should focus on advancing high-resolution, single-cell, and multi-omics technologies to further refine our understanding of these epigenetic echoes and their role in tumor evolution and heterogeneity. Additionally, exploring the therapeutic “sweet spot” for targeting genomic instability and optimizing epigenetic therapies will be essential. By addressing these scientific and ethical challenges, the field can move closer to fully realizing the potential of genomic and epigenomic insights in cancer therapy, offering new hope for more precise and effective treatments.

## Figures and Tables

**Table 1:** Overview of epigenetic markers for early lung cancer detection.

**DNA Methylation Markers**	**RASSF1A (Ras Association Domain Family Member 1):** Frequently hypermethylated in lung cancer. Associated with silencing of tumor suppressor genes, a key marker for early detection [74]. **CDKN2A (Cyclin-Dependent Kinase Inhibitor 2A):** Hypermethylation of the p16 promoter is common in NSCLC [75]. Plays a role in cell cycle regulation. **MGMT (O6-Methylguanine-DNA Methyltransferase):** Hypermethylation silences this DNA repair gene. A potential marker for early-stage lung cancer [76]. **RAR** β **2 (Retinoic Acid Receptor Beta):** Hypermethylation disrupts cell differentiation and increases tumorigenesis [77]. **SHOX2 (Short Stature Homeobox 2):** Validated as a diagnostic biomarker in liquid biopsies for early lung cancer detection [74]. **APC (Adenomatous Polyposis Coli):** Promoter methylation inactivates tumor suppressor pathways in lung cancer [78].
**Non-Coding** **RNA Markers**	**MicroRNAs (miRNAs):** **miR-21:** Overexpressed in lung cancer and other cancer types, associated with poor prognosis [79]. **miR-34a:** Tumor suppressor silenced through promoter methylation, linked to cancer progression [80]. **Long Non-Coding RNAs (lncRNAs):** **MALAT1 (Metastasis-Associated Lung Adenocarcinoma Transcript 1):** Upregulated in lung cancer, implicated in metastasis, and serves as a potential early detection biomarker [81,82].
**Histone** **Marks**	**H3K27me3 (Trimethylation of Histone H3 Lysine 27):** Often associated with gene silencing in lung cancer. Altered patterns may serve as biomarkers for early disease detection [83].
**Liquid Biopsy-** **Based Markers**	**Circulating Tumor DNA (ctDNA):** Epigenetic markers in ctDNA, exosomes, or sputum are minimally invasive options [84]. **Gene Panels:** RASSF1A, SHOX2, and p16 methylation validated for lung cancer screening [85]. **Methylation Signatures:** Sputum methylation profiles help identify early-stage lung cancer in high-risk populations [86]. **Diagnostic Tools:** **Epi proLung^®^ Test:** Detects methylation of SHOX2 and PTGER4 in plasma or bronchial aspirates [87]. **cfDNA-Based Assays:** Emerging tools for ctDNA methylation analysis in early cancer detection [88].

**Table 2:** Active Clinical Trials Investigating Epigenetic Therapies in Lung Cancer (Information retrieved on 17 January 2025).

Study Characteristics	Mechanism of Action	Therapeutic Focus
NCT03220477 https://clinicaltrials.gov/study/NCT03220477 Mocetinostat Phase I	Mocetinostat will be given as treatment and side effect observation	Pembrolizumab in Combination with Guadecitabine and Mocetinostat for patients with Advanced Lung Cancer
NCT05573035 https://clinicaltrials.gov/study/NCT05573035 LYL845 Phase I	Epigenetically reprogrammed tumor infiltrating lymphocyte therapy	Evaluate the safety and anti-tumor activity of LYL845 in participants with relapsed or refractory metastatic or locally advanced NSCLC
NCT06694454 https://clinicaltrials.gov/study/NCT06694454 AZA-AEGEAN Phase I/II	Inhaled Azacytidine With Platinum-Based Chemotherapy and Durvalumab	Determine the frequency of pathologic complete responses in participants for early-stage NSCLC
NCT02664181 https://clinicaltrials.gov/study/NCT02664181 Tetra-hydrouridine- decitabine (THU-Dec) Phase II	Investigation THU-Dec in combination with Nivolumab	Epigenetic immunotherapy for second line therapy in patients with NSCLC
NCT02546986 https://clinicaltrials.gov/study/NCT02546986 CC-486 Pembrolizumab Phase II	Assess the safety and efficacy of combination therapy	Epigenetic modulation and immune checkpoint therapy
NCT04814407 https://clinicaltrials.gov/study/NCT04814407 ctDNA Observational	Circulating Epigenetic Biomarkers	Identification of novel circulating methylated biomarkers for early lung cancer detection
NCT05707585 https://clinicaltrials.gov/study/NCT05707585 Biopsy Observational	Epigenetic Imprinting Biomarkers	Distinguish benign and malignant pulmonary nodules presurgically
NCT02259218 https://clinicaltrials.gov/study/NCT02259218 Molecular profiling Observational	Identification of predictive biomarkers for radiation toxicity and survival	Collected blood, urine, and tissue samples are analyzed for biomarkers via metabolomic and epigenetic profiling
NCT06717243 https://clinicaltrials.gov/study/NCT06717243 Molecular profiling Observational	Genomic and Epigenetic Markers Associated with Resistance to Chemo-Immuno-therapy	Capture the dynamic changes that occur in the tumor microenvironment and how these relate to treatment outcomes

## Data Availability

No new data were created or analyzed in this study. Data sharing is not applicable to this article.
